# Effect of Solids-To-Liquids, Na_2_SiO_3_-To-NaOH and Curing Temperature on the Palm Oil Boiler Ash (Si + Ca) Geopolymerisation System

**DOI:** 10.3390/ma8052227

**Published:** 2015-04-28

**Authors:** Zarina Yahya, Mohd Mustafa Al Bakri Abdullah, Kamarudin Hussin, Khairul Nizar Ismail, Rafiza Abd Razak, Andrei Victor Sandu

**Affiliations:** 1 Center of Excellence Geopolymer and Green Technology, School of Materials Engineering, Universiti Malaysia Perlis (UniMAP), P.O. Box 77, D/A Pejabat Pos Besar, Kangar 01000, Perlis, Malaysia; E-Mails: vc@unimap.edu.my (K.H.); rafizarazak@unimap.edu.my (R.A.R.); 2 Faculty of Engineering Technology, Uniciti Alam Campus, Universiti Malaysia Perlis, Sungai Chuchuh 02100, Padang Besar, Perlis, Malaysia; 3 Faculty of Technology, Universitas Ubudiyah Indonesia, Jl. Alue Naga, Kec. Syiah Kuala Desa Tibang 23536, Banda Aceh, Indonesia; 4 School of Environmental Engineering, Universiti Malaysia Perlis (UniMAP), P.O. Box 77, D/A Pejabat Pos Besar, Kangar 01000, Perlis, Malaysia; E-Mail: nizar@unimap.edu.my; 5 Faculty of Materials Science and Engineering, Gheorghe Asachi Technical University of Iasi, Blvd. D. Mangeron 41, Iasi 700050, Romania; E-Mail: sav@tuiasi.ro

**Keywords:** POBA, geopolymer, alkaline activator, NaOH, sodium silicate

## Abstract

This paper investigates the effect of the solids-to-liquids (S/L) and Na_2_SiO_3_/NaOH ratios on the production of palm oil boiler ash (POBA) based geopolymer. Sodium silicate and sodium hydroxide (NaOH) solution were used as alkaline activator with a NaOH concentration of 14 M. The geopolymer samples were prepared with different S/L ratios (0.5, 1.0, 1.25, 1.5, and 1.75) and Na_2_SiO_3_/NaOH ratios (0.5, 1.0, 1.5, 2.0, 2.5, and 3.0). The main evaluation techniques in this study were compressive strength, X-Ray Diffraction (XRD), Fourier Transform Infrared Spectroscopy (FTIR), and Scanning Electron Microscope (SEM). The results showed that the maximum compressive strength (11.9 MPa) was obtained at a S/L ratio and Na_2_SiO_3_/NaOH ratio of 1.5 and 2.5 at seven days of testing.

## 1. Introduction

The study of alkali-activated binder was started by Purdon in the 1940s in which blast furnace slag was activated with sodium hydroxide (NaOH) solution [1]. After that, in the late 1950s and 1960s Glukhovsky invented an alkali activated system which contained calcium silicate hydrate (CSH) and aluminosilicate phases [2]. Besides that, the term geopolymer was introduced by Davidovits in 1972 where it was described as tri-dimensional alumina-silicate which formed at low temperature and short time from naturally occurring alumina-silicates such as kaolin [3]. Geopolymerisation is a geosynthesis where the reaction integrates minerals from alumino-silicate sources [4,5] and an exothermic process is involved. Any raw material rich in silica and alumina or pozzolanic materials which can be dissolved in an alkaline activator solution can hence undergo a geopolymerisation process.

Geopolymer systems can be divided into two types of binding system which are silica-aluminum (Si + Al) with medium to high alkaline solution and silica-calcium (Si + Ca) with a mild alkaline solution [6]. For the (Si + Al) binding system, the source materials included in this system are class F fly ash and metakaolin due to having silica and alumina content as the main composition. Meanwhile, for the (Si + Ca) system, ground granulated blast furnace slag (GGBS) was included in this system due to its main composition which is silica and calcium. The hydration products of these two systems are also different where for the (Si + Ca) system, calcium silicate hydrate (CSH) is the main product and zeolite like polymers are the main products for the (Si + Al) system.

For the alkaline activator solution, the combination of NaOH and sodium silicate solution (Na_2_SiO_3_) leads to higher geopolymerisation rates compared to hydroxide alone [6]. Moreover, it was proved by Xu and Van Deventer [7] where different source materials of alumina-silicate mineral are used to produce geopolymer, it required additional silica (Si) for the geopolymerisation process. Alkali hydroxide is required for the dissolution process of aluminosilicate sources, while Na_2_SiO_3_ solution acts as binder [2].

The mix design for geopolymers can be divided into solids/liquid (S/L) and sodium silicate/NaOH (Na_2_SiO_3_/NaOH) ratio which is important in developing the mechanical strength of the geopolymer [6,8]. Fresh geopolymer paste with a high S/L ratio had a low viscosity while geopolymer paste with a low S/L ratio resulted in high viscosity [9]. Besides that, geopolymer with a low S/L ratio could accelerate the dissolution rate, but it was not applicable to the polycondensation process when high concentrations of NaOH were used [10]. In addition, the low S/L ratio contributed to low strength due to insufficient formation of binder [11].

The influence of the Na_2_SiO_3_/NaOH ratio (0.4, 1.5, 5.0, 10.0, and 15.0) in natural zeolite based geopolymers showed that increasing the Na_2_SiO_3_/NaOH ratio up to 1.5 increased the compressive strength, but beyond that the strength was decreased [12]. This may be due to excessive sodium silicate that retarded the geopolymerisation process by the precipitation of Al-Si phase, which prevented contact between the reacting material and activating solution and decreased the activator content [13]. Researchers have suggested that the optimum Na_2_SiO_3_/NaOH ratio to produce high strength geopolymer is in the range 0.67–1.00 [14]. Meanwhile, Hardjito* et al.* [15] investigated the effect of two different Na_2_SiO_3_/NaOH ratios (0.4 and 2.5) on the performance of fly ash based geopolymer. They found that when the ratio increased the strength of geopolymer also increased.

Malaysia is one of the world’s largest producers of palm oil products and as such the waste material from this industry is also widely available and estimated to be about 44.9 million tonnes. The solid wastes are burned in the boiler to generate electricity at the palm oil mill and the palm oil boiler ash (POBA) or bottom ash is obtained at the lower compartment of the boiler. Generally, palm oil fuel ash (POFA) is used as a cement replacement in concrete as POBA contains coarse particles. As such this study investigates the utilization of POBA in geopolymers and the effect of solids-to-liquids ratio (S/L), alkaline activator ratio (Na_2_SiO_3_/NaOH) and curing temperature on geopolymer paste. The results of the geopolymer paste are evaluated in terms of compressive strength, X-Ray Diffraction (XRD), Fourier Transform Infrared Spectroscopy (FTIR), and Scanning Electron Microscope (SEM).

## 2. Results and Discussion

### 2.1. Compressive Strength 

**Figure 1:**
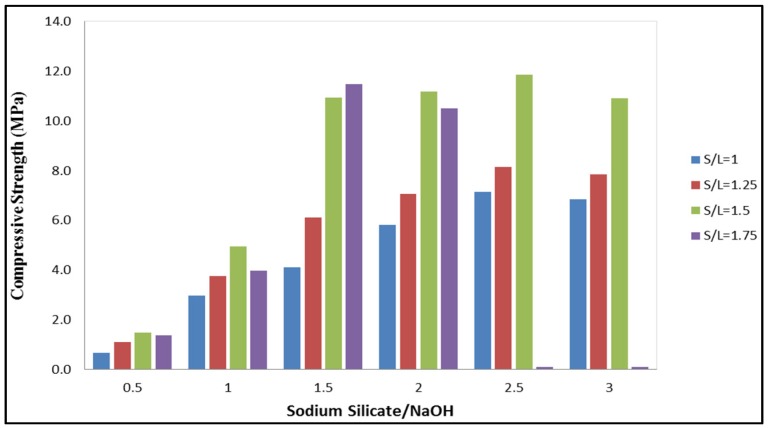
Compressive strength of geopolymer samples with different solid/liquid (S/L) and Na_2_SiO_3_/NaOH ratios at 7 days.

The strength of the geopolymer paste with different solid/liquid (S/L) and Na_2_SiO_3_/NaOH ratios is shown in Figure 1. When the S/L and Na_2_SiO_3_/NaOH ratio increased, the compressive strength also increased. The maximum compressive strength (11.9 MPa) was achieved at S/L and Na_2_SiO_3_/NaOH ratio of 1.5 and 2.5. Furthermore, at S/L ratio 1.0, 1.25 and 1.5 the maximum compressive strength was obtained at a Na_2_SiO_3_/NaOH ratio of 2.5. The compressive strength of geopolymer paste increased when the amount of Na_2_SiO_3_ increased. Moreover, the use of Na_2_SiO_3_ helps to improve the geopolymerisation process by accelerating the dissolution of source material [7]. It was observed by Hardjito and Rangan [16] that increasing the Na_2_SiO_3_/NaOH ratio increased the geopolymerisation rate. Conversely, when the Na_2_SiO_3_/NaOH ratio was more than 3.0 the compressive strength tended to decrease for all S/L ratios. This may be due to excessive alkali content which retards the geopolymerisation process. It occurs when Al-Si phase precipitation prevents interaction between reacting material and alkaline activator thus reducing the activator concentration [12].

However, at S/L ratio 1.75 with Na_2_SiO_3_/NaOH ratios 2.5 and 3.0 the geopolymer paste was unable to mix due to lower workability. As such, geopolymer strength was unable to be acquired. This condition showed that the Na_2_SiO_3_/NaOH ratio was correlated to the workability and compressive strength of the geopolymer paste. For this S/L ratio, the maximum strength (11.5 MPa) was contributed by a geopolymer sample with a Na_2_SiO_3_/NaOH ratio 1.5.

Meanwhile, for S/L ratio 1.0 and 1.25 contributed a low strength of geopolymer paste and can be due to a high content of alkaline activator. The high content of alkaline activator produced excessive OH^−^ that was left in the system, thus weakening the geopolymer structure [17]. From this study it was shown that S/L and Na_2_SiO_3_/NaOH ratios did influence the compressive strength and the workability of geopolymers. The optimum mix design for POBA geopolymer paste was obtained at S/L and Na_2_SiO_3_/NaOH ratios of 1.5 and 2.5, respectively.

**Figure 2:**
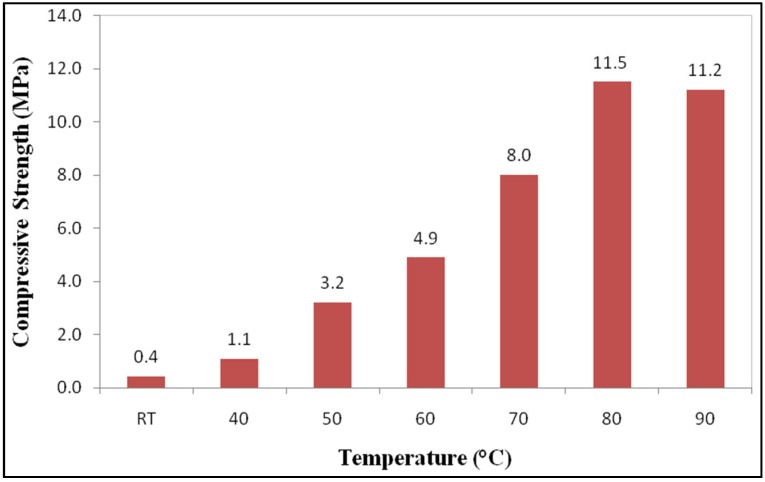
Compressive strength of geopolymer with different curing temperatures at 7 days.

Figure 2 shows the effect of curing temperature on the compressive strength of geopolymer paste where at room temperature (RT) the lowest compressive strength (0.4 MPa) was obtained. Meanwhile, the maximum compressive strength (11.5 MPa) was obtained at a curing temperature 80 °C for 24 h. When the curing temperature increased, the compressive strength also resulted in an increment. The geopolymer samples displayed increment in strength in the range 3%–96% when cured at different temperatures. Hence, from this result it is indicated that geopolymer paste produced using POBA, required heat curing in order to increase the compressive strength. Palomo* et al.* [6] also mentioned that heat curing acts as an accelerator in the geopolymer production. Heat curing in geopolymers leads to a quicker geopolymerisation process, thus producing adequate strength within a very short period [16,18,19,20].

Geopolymer samples exhibited slightly reduced compressive strength after curing at temperature 90 °C. Bakharev [21] found that geopolymer samples cured at 90 °C experienced a significant loss of moisture. As such, Hardjito* et al.* [17] concluded that curing samples at higher temperature does not essentially produce higher strength geopolymer products. Moreover, since the geopolymer samples that were produced using a 50mm mould had a high surface-to-volume ratio, this is more vulnerable to heat curing and also loss of moisture, and thus could lead to strength reduction when cured at high temperature [14]. Additionally, Chindaprasirt* et al.* [14] stated that to produce geopolymer with good strength requires the presence of moisture.

### 2.2. X-Ray Diffraction (XRD) Analysis

**Figure 3:**
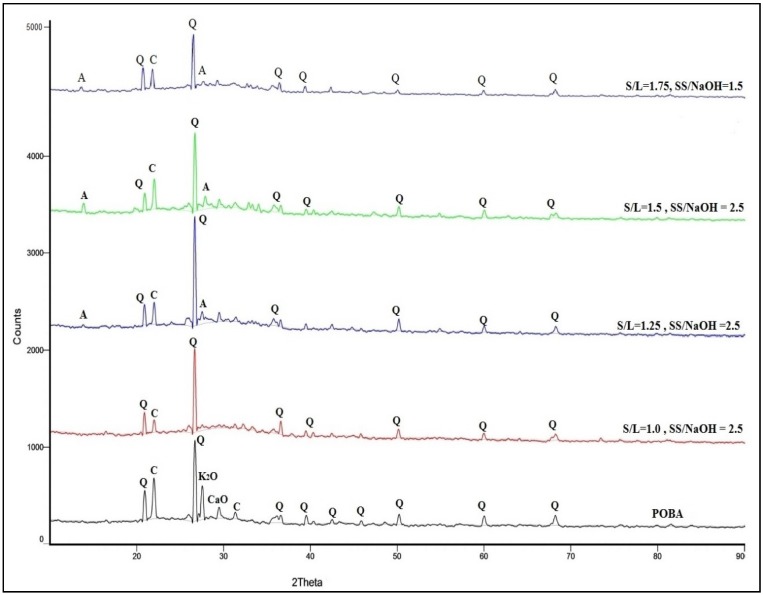
X-Ray Diffraction (XRD) analysis on geopolymer paste with different mix design (Q = quartz, C = cristobalite, A = albite, K_2_O = potassium oxide, CaO = calcium oxide, C = calcium).

The X-Ray Diffraction (XRD) analysis for S/L of 1.0, 1.25, 1.5, and 1.75 with maximum compressive strength is presented in Figure 3. From the figure it is shown that the highest peak for all mix design was contributed by quartz. At ratio S/L 1.0, only quartz and cristobalite peaks were detected compared to S/L 1.25, 1.5, and 1.75. However, at ratio S/L 1.25, 1.5 and 1.75 peaks of albite were found at 2θ = 13° and 2θ = 28°, approximately. The highest peak of albite was contributed by S/L 1.5 which is consistent with optimum compressive strength. The peak of albite was attributed to the strength of the geopolymer paste by forming a crystalline phase of the N-A-S-H (aluminosilicate gel) system [22].

The peaks of cristobalite and quartz at 2θ = 21° and 2θ = 22° in POBA still exists in geopolymer paste. Besides that, the geopolymer paste with the different mix design also demonstrated an amorphous to semi crystalline phase which is the same as with POBA. Since the crystalline peaks were detected more with a S/L ratio of 1.5, it can be concluded that the existence of these peaks helps to increase the strength of the geopolymer paste [23].

**Figure 4:**
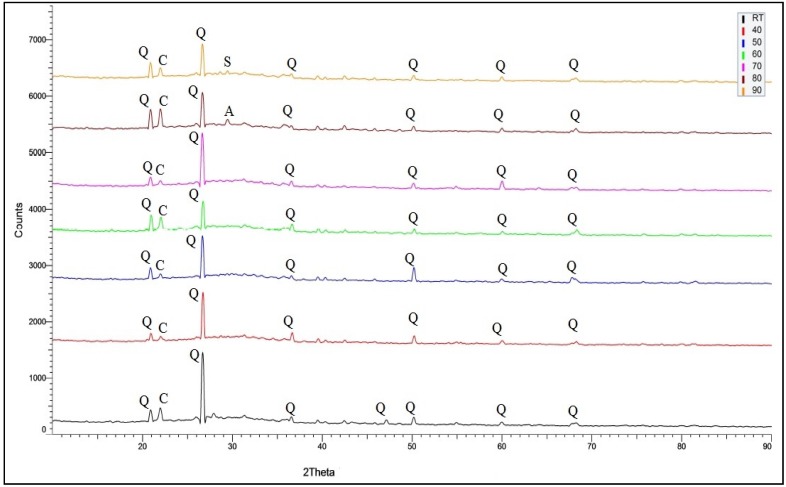
X-Ray Diffraction (XRD) analysis on geopolymer paste with different curing temperatures (Q = quartz, C = cristobalite, A = albite, S = sodium silicate).

Figure 4 demonstrates the XRD analysis on geopolymer samples cured with different curing temperatures for 24 h. Quartz and cristobalite were spotted in all samples with maximum peaks of quartz. All the geopolymer samples still displayed the amorphous phase despite being cured at different temperatures. The remaining quartz and cristobalite in geopolymer samples showed that the originally used quantity had not fully reacted during the geopolymerisation process for all temperatures.

### 2.3. Fourier Transform Infrared Spectroscopy (FTIR) Analysis 

**Figure 5:**
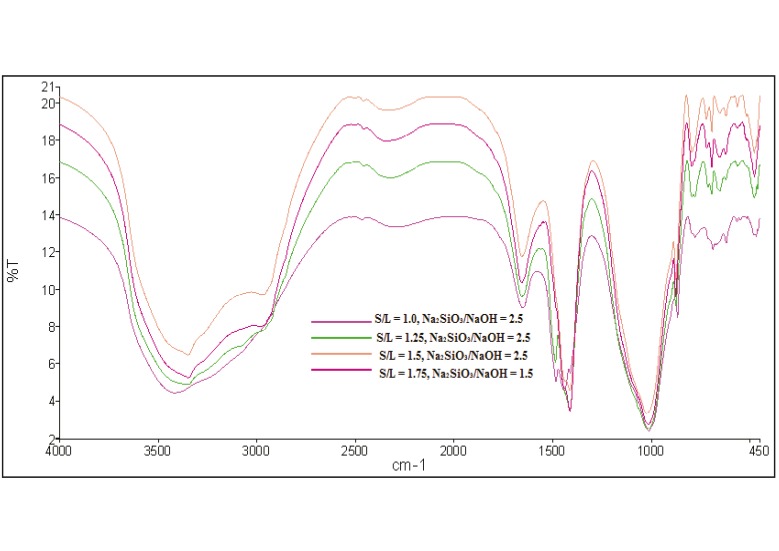
IR spectra of geopolymer paste with different mix design.

Figure 5 displays the IR spectra of geopolymer samples with the different mix design of S/L ratio. The geopolymer samples with optimum strength at each S/L ratio were analyzed in this study. The broad bands appearing at 2316–3351 cm^−1^ were due to stretching vibrations OH and HOH. In addition, the bending vibration of HOH was detected at 1651–1655 cm^−1^. The existence of these bondings was related to entrapped water molecules in the geopolymeric network.

The stretching vibration of O-C-O was still detected in each of the geopolymer samples at 1411–1412 cm^−1^[23] which was attributed to the carbonation reaction. The carbonation process occurred because an excessive amount of Na was available from the alkaline activator solution where it reacted with CO_2_ from the atmosphere [24].

The band attributed to asymmetric stretching vibration of Si-O-Si and Al-O-Si around area 1011–1027 cm^−1^ indicated the formation of aluminosilicate gel [25]. Besides that, symmetric stretching vibrations Si-O-Si were located at 778–795 cm^−1^. At 583–721 cm^−1^, symmetric stretching vibrations of Si-O-Si and Al-O-Si were identified. In the meantime, the bending vibrations of Si-O-Si and O-Si-O were found at area 467–479 cm^−1^.

**Figure 6:**
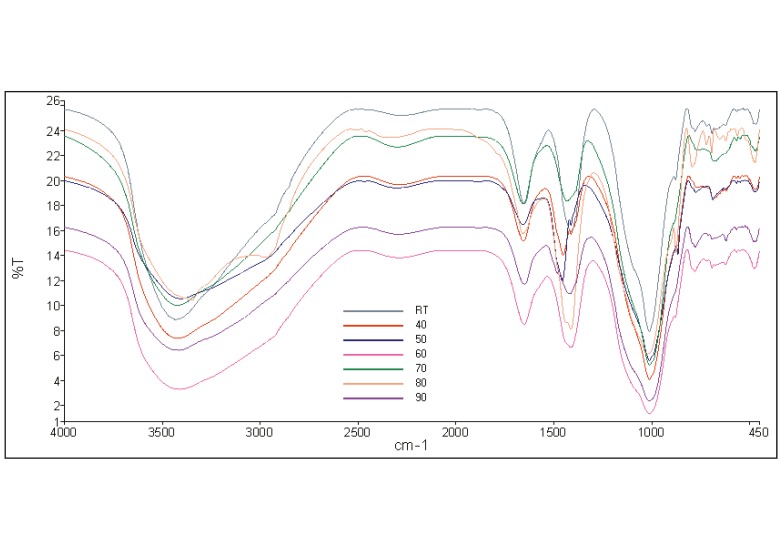
IR spectra of geopolymer paste with different curing temperatures.

The broad band appeared in all IR spectra in the region 2287–3435 cm^−1^ indicating the presence of stretching vibrations OH and HOH as in Figure 6. Meanwhile bending vibration HOH was detected at 1648–1658 cm^−1^ where all these bondings represent water molecule. The band at 1412–1432 cm^−1^ represents the stretching vibration of O-C-O. The aluminosilicate gel (asymmetric stretching vibrations Si-O-Si and Al-O-Si) were detected at 1011–1023 cm^−1^. When the curing temperature increased, the asymmetric stretching vibrations Si-O-Si and Al-O-Si also shift to lower frequency. Besides that, the symmetric stretching vibration Si-O-Si was found at 776–794 cm^−1^.

In addition, the symmetric stretching vibrations Si-O-Si and Al-O-Si were located at 621–721 cm^−1^. However, at 467–476 cm^−1^, bending vibrations Si-O-Si and O-Si-O were identified which represents quartz, thus indicating that quartz which originally exists in POBA did not fully react with the alkaline activator solution which correlates with the finding in XRD analysis.

### 2.4. Scanning Electron Microscope (SEM) Analysis

**Figure 7:**
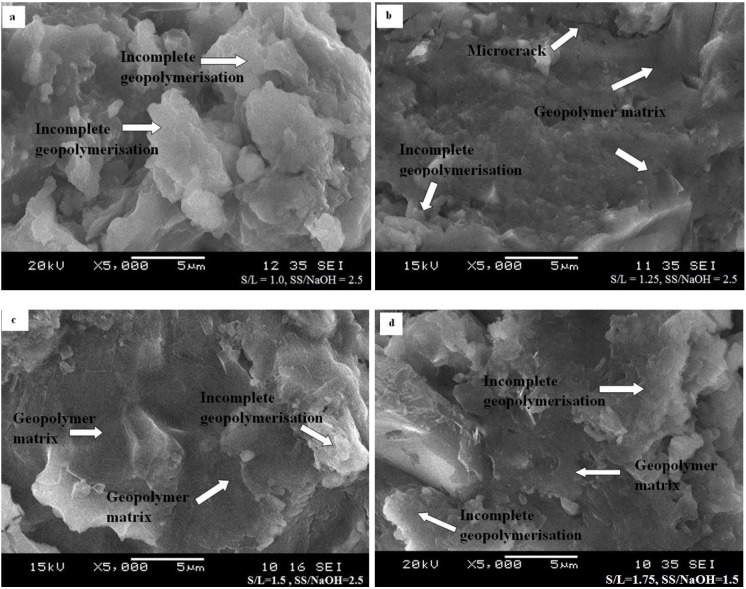
Geopolymer with different mix design. (**a**)S/L = 1.0, Na_2_SiO_3_/NaOH=2.5; (**b**) S/L = 1.25, Na_2_SiO_3_/NaOH=2.5; (**c**) S/L = 1.5, Na_2_SiO_3_/NaOH=2.5; (**d**) S/L = 1.75, Na_2_SiO_3_/NaOH=1.5.

For each S/L ratio (1.0, 1.25, 1.5, and 1.75) the samples that contributed the maximum compressive strength are displayed in Figure 7a–d. Geopolymer samples with ratio S/L 1.0 and Na_2_SiO_3_/NaOH 2.5 are shown in Figure 7a where it demonstrates incomplete geopolymerisation. This leads to a less dense geopolymer matrix and lower compressive strength (7.2 MPa). Since the quantity of POBA and alkaline activator was equal in this sample, it takes time for the geopolymerisation process to complete.

Besides that, Figure 7b with ratio S/L 1.25 and Na_2_SiO_3_/NaOH 2.5 demonstrated a denser geopolymer matrix compared to previous figures. Therefore, the quantity of alkaline activator affects the saturation rate of the geopolymerisation process and the strength of geopolymer. In this sample, microcrack was detected and may be due to the sample preparation for the SEM analysis.

Geopolymer samples with ratio S/L 1.25 and Na_2_SiO_3_/NaOH 2.5 as in Figure 7c show a denser geopolymer matrix compared to others. It shows POBA reacts homogeneously with alkaline activator thus leading to maximum compressive strength (11.9 MPa). Nevertheless, incomplete geopolymerisation is still observed in this sample.

Figure 7d illustrates a geopolymer sample with ratio S/L 1.75 and Na_2_SiO_3_/NaOH 1.5 and the maximum compressive strength (11.5 MPa) was obtained. For this mix design with Na_2_SiO_3_/NaOH more than 2.0, the geopolymer samples were unable to be prepared due to low workability. As such, from the figure it shows incomplete geopolymerisation due to less alkaline activator available to react with POBA.

**Figure 8:**
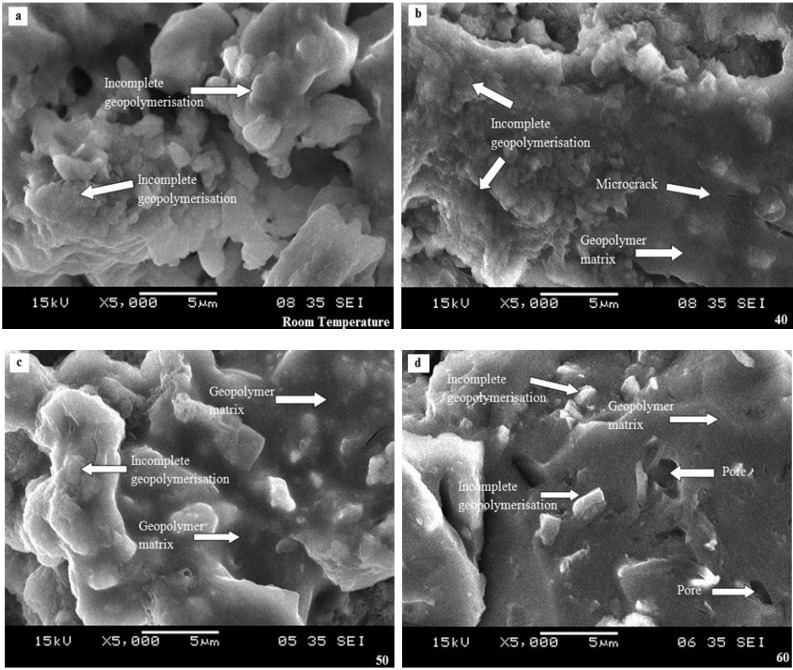

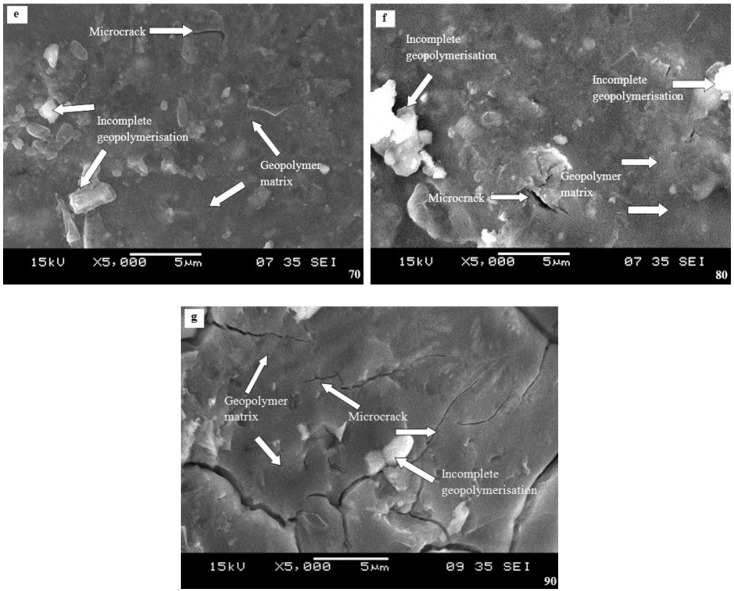
Geopolymer samples with different curing temperatures. (**a**) Room Temperature; (**b**) 40 °C; (**c**) 50 °C; (**d**) 60 °C; (**e**) 70 °C; (**f**) 80 °C; (**g**) 90 °C.

The morphology of geopolymer samples with different curing temperature is displayed in Figure 8a–g. Geopolymer cured at room temperature (Figure 8a) displayed more incomplete geopolymerisation compared to other samples. The reaction rate between POBA and alkaline activator occurred very slowly where no solid geopolymer matrix is formed. Subsequently, due to a slow geopolymerisation process, the strength was also low.

In the meantime, a geopolymer sample cured at 40 °C showed less incomplete geopolymerisation and a dense matrix was formed. At this temperature, the geopolymerisation process starts only slowly to form the geopolymer matrix. A dense gel-like matrix imbedded with POBA particles is seen in Figure 8b. Besides that, the microcrack detected was due to sample preparation for morphological analysis. In Figure 8c, the hardening process in the geopolymerisation covered POBA particles with a dense gel-like matrix. The unreacted POBA particles were seen on the surface of the dense geopolymer matrix. Jaarsveld* et al.* [26] mentioned that the dissolution of source material is not complete where in many cases the surface reaction is responsible for the formation of the geopolymer final structure in bonding the undissolved particles.

At a curing temperature of 60 °C (Figure 8d), a dense geopolymer matrix was observed with some unreacted POBA particles. The existence of pores was also detected in this sample. Since a more dense geopolymer structure was produced, the strength also increased. For geopolymer samples cured at 70 °C, 80 °C, and 90 °C, dense geopolymer matrix was produced as well as maximum compressive strength at 80 °C. The geopolymerisation process completely occurred when the geopolymer sample was cured at 70 °C. However, many microcracks were observed in the geopolymer sample cured at 90 °C. This may be due to the high curing temperature that causes a quick hardening process thus leading to microcracks. The strength also reduces when cured at high temperature.

## 3. Experimental Section 

### 3.1. Material

**Figure 9:**
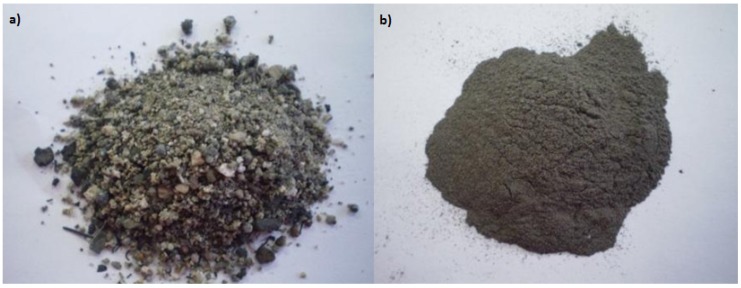
(**a**) Palm oil boiler ash (POBA); (**b**) fine POBA.

The POBA was obtained from United Palm Oil Mill in Penang, Malaysia where it contained large particles which included unburned nutshells, fibers, and kernels as in Figure 9a. Then the POBA was ground using a heavy duty grinder in order to obtain finer particles. After that, the ground POBA was sieved using 100 µm sieves. The POBA that passed through the 100 µm sieve (Figure 9b) was used to produce geopolymer paste and the chemical composition is as in Table 1 below [27]. The POBA was classified as a silica-calcium (Si + Ca) geopolymerisation system due to the high content of silica (Si) and calcium (Ca).

**Table 1:** Chemical composition of fine palm oil boiler ash (POBA).

No.	Compositions	POBA (wt%)
1	SiO_2_	40.60
2	Al_2_O_3_	3.71
3	Fe_2_O_3_	15.74
4	CaO	19.60
5	MgO	1.30
6	P_2_O_5_	2.73
7	K_2_O	13.80
8	SO_3_	0.44
9	TiO_2_	0.35
10	MnO	0.28

NaOH solution and sodium silicate (Na_2_SiO_3_) solution were used as alkaline activator to synthesize POBA. NaOH pellets with 99% purity brand name of Formosoda-P, from the Formosa Plastic Corporation, Taiwan were used to produce the NaOH solution. The NaOH solution with 14 M concentration was prepared by diluting NaOH pellets with distilled water. Sodium silicate (Na_2_SiO_3_) solution was obtained from South Pacific Chemical Industries Sdn. Bhd. (SPCI), Malaysia.

### 3.2. Mix Design 

#### 3.2.1. Solids-to-Liquids (S/L) and Na_2_SiO_3_/NaOH

The fine POBA was mixed with an alkaline activator with four different S/L (POBA/alkaline activator) ratios such as 1.0, 1.25, 1.5, and 1.75. Meanwhile, for alkaline activator solution (Na_2_SiO_3_/NaOH ratio), six solutions were prepared according to the ratio 0.5, 1.0, 1.5, 2.0, 2.5, and 3.0 [28,29,30,31]. Table 2 shows the experimental details for solid/liquid ratios and Na_2_SiO_3_/ NaOH ratios. All the samples were cured at 80 °C for 24 h and left at room temperature for 7 days for compressive strength testing.

#### 3.2.2. Various Curing Temperatures

After the optimum S/L ratio and Na_2_SiO_3_/NaOH was obtained, further study was conducted to investigate the effect of curing temperature. The samples were cured at room temperature (RT), 40 °C, 50 °C, 60 °C, 70 °C, 80 °C, and 90 °C for 24 h [32]. Then, all the samples were cured at room temperature for 7 days for compressive strength testing.

**Table 2:** Mix design details for geopolymer pastes.

S/L Ratio	Na_2_SiO_3_/NaOH Ratio	Mass of Solid (g)	Na_2_SiO_3_ Solution (g)	NaOH Solution (g)
1.0	0.5	480	160.0	320.0
	1.0		240.0	240.0
	1.5		288.0	192.0
	2.0		320.0	160.0
	2.5		342.9	137.1
	3.0		360.0	120.0
1.25	0.5	480	128.0	256.0
	1.0		192.0	192.0
	1.5		230.4	153.6
	2.0		256.0	128.0
	2.5		274.3	109.7
	3.0		288.0	96.0
1.5	0.5	480	106.7	213.3
	1.0		160.0	160.0
	1.5		192.0	128.0
	2.0		213.3	106.7
	2.5		228.6	91.4
	3.0		240.0	80.0
1.75	0.5	480	91.4	182.9
	1.0		137.1	137.1
	1.5		164.6	109.7
	2.0		182.9	91.4
	2.5		195.9	78.4
	3.0		205.7	68.6

### 3.3. Mixing Process

The alkaline activator solution was prepared by mixing NaOH solution with Na_2_SiO_3_ solution until a homogeneous solution was achieved. Then, the alkaline activator was mixed with POBA in the mechanical mixer for about 5 min approximately. The geopolymer paste was placed in a mould (50 × 50 × 50 mm) and then placed in a vibrating table for 10 s to remove entrapped air. The geopolymer samples that underwent heat curing were covered with a plastic sheet to avoid moisture loss.

### 3.4. Testing

#### 3.4.1. Compressive Strength 

The strength of geopolymer pastes was measured using compressive strength testing based on American Society for Testing and Materials (ASTM C109). The testing was carried out using an Instron machine series 5569 Mechanical Tester (Instron, Singapore) with maximum loading 50 KN and speed rate 50 mm/min. Three samples were used for each mix design to determine the optimum compressive strength.

#### 3.4.2. X-Ray Diffraction (XRD)

The phase of geopolymer paste that leads to maximum compressive strength was determined using XRD. The geopolymer paste was crushed into powder and tested using a XRD-6000, Shimadzu X-ray diffractometer using Cu-Kα radiation generated at 30 Ma and 40 kV. The samples were tested in powder form starting from 10° to 90° (2θ) at 0.04° steps with step time 1.0 s.

#### 3.4.3. Fourier Transform Infrared Spectroscopy (FTIR)

The FTIR analysis was conducted using a Perkin Elmer FTIR Spectrum RX1 Spectrometer. The samples of geopolymer paste were prepared in powder form where they were mixed with potassium bromide (KBr), then a cold press machine was used with a 4 ton loading for 2 min. All the samples used wavelengths from 450 cm^−1^ to 4000 cm^−1^.

#### 3.4.4. Microstructure Analysis

The microstructure of POBA geopolymer paste was observed using Scanning Electron Microscope (SEM). The geopolymer paste samples were cut into small pieces and coated with platinum by using an Auto Fine Coater. A JSM-6460LA model Scanning Electron Microscope (JEOL, Pleasanton, CA, USA) was used in this analysis.

## 4. Conclusions

From this study the results led to the conclusions below:

(a) The optimum mix design for geopolymer paste using POBA is S/L = 1.5 and Na_2_SiO_3_/NaOH = 2.5 with maximum compressive strength 11.9 MPa. During XRD analysis, the existence of albite which is due to the formation of an aluminosilicate gel was detected in the optimum mix design. The ratio of Na_2_SiO_3_/NaOH also plays an important role in the mix design of the geopolymer paste. When the ratio of Na_2_SiO_3_/NaOH is more than 2.5, the compressive strength for S/L (1.0, 1.25, and 1.5) tends to decrease due to excessive alkali content that retards the geopolymerisation process.
(b) The optimum curing temperature for POBA in this study was 80 °C which led to maximum compressive strength (11.5 MPa) at 24 h curing period. Thus, heat curing for geopolymer is needed in order to obtain sufficient strength and with heat curing, the geopolymerisation becomes more rapid. The presence of albite during XRD analysis was also detected in the geopolymer sample cured at 80 °C. The morphology of the geopolymer samples showed changes in the matrix when the curing temperature was increased.
